# Case Report: Molecular characterization of rabies virus transmitted from a dog to a bull in a livestock market in Ghana

**DOI:** 10.3389/fvets.2025.1524562

**Published:** 2025-06-18

**Authors:** Theophilus Odoom, Richard Kwamena Abbiw, Joseph Kofi Abuh, Emmanuel Allegye-Cudjoe, Sherry Ama Mawuko Johnson, William Tasiame, Daniel Arthur, Benita Anderson, Tirumala B. K. Settypalli, Charles E. Lamien, William G. Dundon

**Affiliations:** ^1^Accra Veterinary Laboratory, Veterinary Service Directorate, Accra, Ghana; ^2^School of Veterinary Medicine, CBAS, University of Ghana, Accra, Ghana; ^3^West African Centre for Cell Biology of Infectious Pathogens, University of Ghana, Accra, Ghana; ^4^Veterinary Service Directorate, Accra, Ghana; ^5^School of Veterinary Medicine, Kwame Nkrumah University of Science and Technology, Kumasi, Ghana; ^6^Animal Production and Health Laboratory, Animal Production and Health Section, Joint FAO/IAEA Center, Department of Nuclear Sciences and Applications, International Atomic Energy Agency, Vienna, Austria

**Keywords:** rabies, bull, dog, Ghana, Africa 2 lineage, phylogeny

## Abstract

On the 24/09/2023, a video of a suspected rabid bull at a livestock market in Ghana was shared on social media and was seen by a local veterinary officer. This led to an on-site investigation by veterinary authorities on the 25/09/2023 which concluded that the bull had been bitten by a three-month old dog 4 days previously. The puppy, which was killed and buried after the bite, was subsequently exhumed, tested and confirmed positive for rabies. The bull was humanely destroyed. Brain tissue from the bull was collected and sent to the Accra Veterinary Laboratory for further analysis. RABV was confirmed by conventional RT-PCR and the full genome of the viruses from both animals were sequenced. The consensus sequences of the genomes belonging to the Africa 2 clade, were identical although sub-consensus variants in a subset of the sequences located in the RNA-dependent-RNA polymerase (L) gene of the bovine virus were observed. The implication of these findings is discussed.

## 1 Introduction

Rabies is a zoonotic disease caused by rabies virus (RABV) which is transmitted by the bite of infected mammals resulting in acute and generally fatal encephalitis (1). Molecular epidemiological studies have demonstrated that RABVs can be divided into dog and bat derived clusters, and subdivided into several clades, subclades and lineages (2). Dog-derived isolates belong to six well defined clades; Cosmopolitan (Africa-1 and Africa-4), Africa-2 and Africa-3 clades have been shown to be present in Africa (2, 3). Africa-1a, Africa-1b and Africa-1c are lineages of Africa-1 (4). Globally, nearly 60,000 humans die of rabies (5, 6). Dogs are integral to the transmission of RABV contributing to 9 of 10 human rabies cases. However, the virus can also infect other mammals including livestock (4–7) and wildlife (7, 8). Therefore, the impact of rabies is not limited to the loss of human lives, but poses palpable threats to livestock production and wildlife surveillance, especially in endemic areas (9–13). RABV is enzootic/endemic in Ghana and has been reported in both humans and animals including dogs, cats, pigs, goats, monkeys, sheep, bats and cattle (14–17). RABV belonging to the Africa 2 lineage and Africa 1 (a and b) sub-lineages have been identified in rabies outbreaks in Ghana (18, 19).

This case report describes the transmission of rabies virus from a puppy to a bull in a livestock market in Ghana. It provides details on the intervention of the veterinary authorities, laboratory diagnosis and molecular characterization of the causative virus. It has provided a unique opportunity to characterise and analyse epidemiologically related RABVs.

## 2 Case description

**Figure 1:**
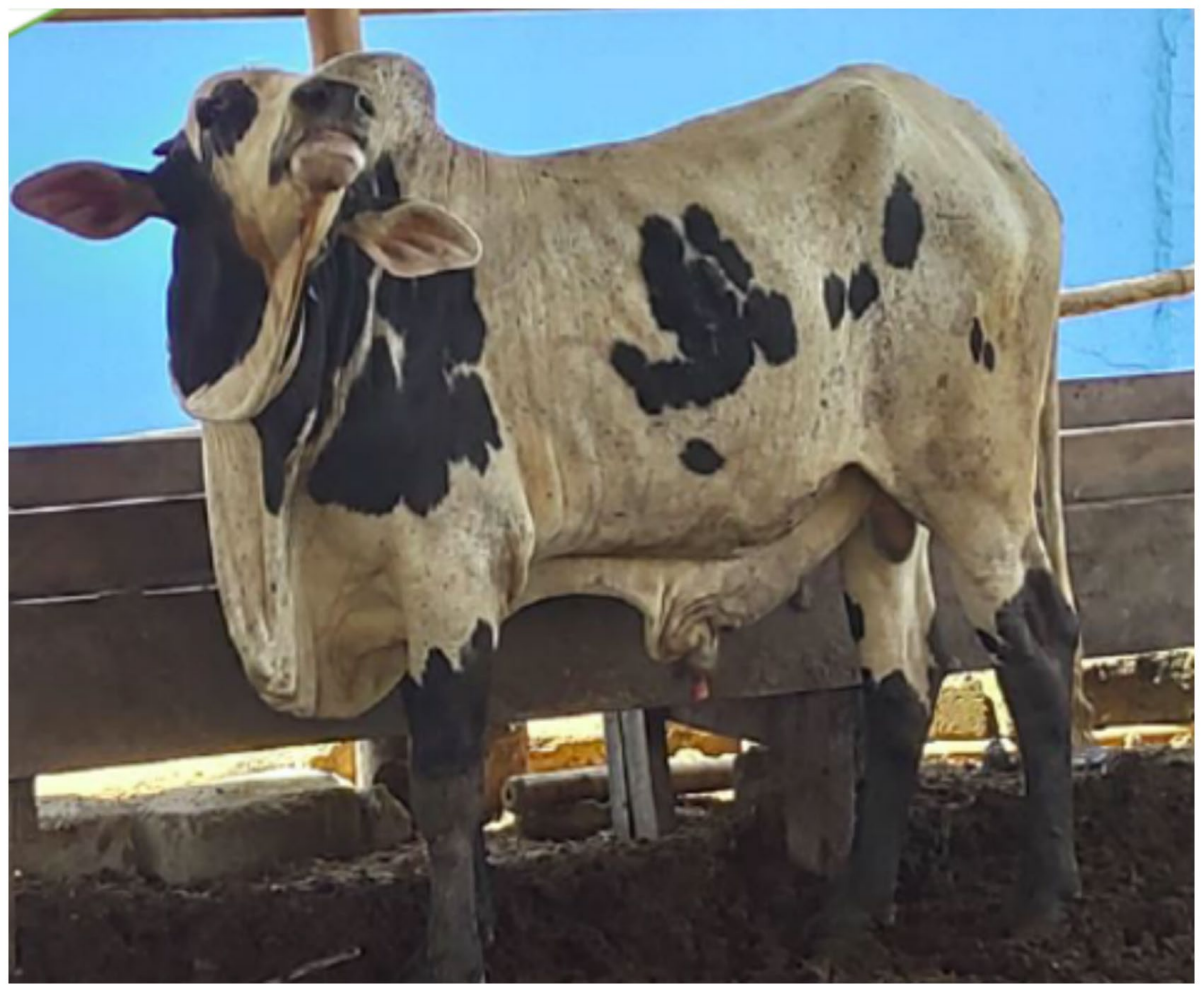
Photograph of the infected bull, Joma livestock market, Accra Ghana.

A video of a suspected rabid bull was seen by the Greater Accra Regional Veterinary Officer (RVO) on the 24/09/2023 (Supplementary Video S1). He immediately informed the Chief Veterinary Officer (CVO) and the Head of the Epidemiology Unit of the Veterinary Service Directorate (VSD) who later led a team of three VSD staff and the District Veterinary Officer (DVO) of the Weija-Gbawe Municipal to investigate the video taken at the Joma livestock market in Ablekuma. The team visited the site a day after the video was taken. According to the investigation, the bull (Figure 1) was found restless and profusely drooling; signs that baffled the owners and led to the videoing of the incident. It was revealed that the bull had been bitten on its nose by a three-month old unvaccinated male puppy on the 22/092023 while resting in a pen at the livestock market with 21 other cattle, 20 sheep, one goat and one horse. The dog was reported to have been overly aggressive, barking incessantly and attempted to bite people but failed. Due to the suspicion of rabies, the dog was captured, killed and buried on the 23/09/2023.

The buried puppy was exhumed (Supplementary Figure S1) and the brain sampled and tested for rabies using an Anigen Rapid Rabies Antigen Test Kit (Bionote Inc., Korea) on 25/09/2023. The bull was quarantined pending confirmatory laboratory diagnosis of the puppy’s brain sample. On 26/09/2024, the bull was euthanised following RT-PCR confirmation of RABV in the puppy. Contact tracing by the team identified 12 people that had come into contact with the suspected rabid animals. These individuals were directed to go for anti-rabies post exposure vaccines, while the residents and workers at the market were educated on rabies from the 25/09/20230 to 27/09/2023. The DVO was tasked by the RVO to provide regular education on rabies.

## 3 Diagnostic assessment

### 3.1 RDT

An Antigen Rapid Rabies Ag Test Kit (Bionote Inc., Korea) was used for the preliminary diagnosis of rabies in both the puppy and the bull post-mortem. The immunochromatographic assay is reported to be 96.9% sensitive and 100% specific for rabies virus. Briefly, 10% brain homogenates prepared in the PBS supplied in the kit was added to the test device and the result was read after 10 min.

### 3.2 RNA extraction

Two grams (2 g) of brain tissues from the bull (C2) and the puppy (D2) were homogenized in a sterile 1.5 mL Eppendorf tube. A second brain sample (D1) from a dog collected on the 28/07/2023 with reference number R/24/19 and confirmed to be positive for rabies was included for comparative purposes. Total viral RNA of the brain samples was extracted using the Qiagen RNeasy RNA mini kit according to the manufacturer’s instructions and stored at −80°C until required. The RNA extracts were used for RT-PCR at the Accra Veterinary Laboratory (AVL) and sequencing at the Animal Production and Health Laboratory, Joint FAO/IAEA Centre, Austria, for full genome sequencing.

### 3.3 RT-PCR

RNA samples were screened at the AVL as described by Benedictis et al. (20) using a one-step RT-PCR kit (Qiagen). Briefly, 2.5 μL of RNA was added to a master mix containing primers RabPyro Forward primer: 5′ – AACACYYCTACAATGGA – 3′ (61.4 nmol), RabPyro reverse primer 1: 5′ - TCCAATTNGCACACATTTTGTG – 3′ (66.1 nmol), RabPyro reverse primer 2: 5′ – TCCARTTAGCGCACAT YTTATG – 3′ (100.8 nmol), and RabPyro reverse primer 3: 5′ – TCCAGTTGGCRCACATCTTRTG – 3′ (97.7 nmol), 1 X RT-PCR buffer, dNTPs (10 mmol), RNase Inhibitor (1 U), enzyme mix (1 u) and RNase free water. The following cycling conditions were used: 50°C, for 30 min; 95°C for 15 min followed by 45 cycles of 94°C for 30 s, 52°C for 30 s and 72°C for 40 s with a final elongation of 72°C for 5 min. Amplicons were visualised on 1.5% (v/v) agarose gels.

### 3.4 Full genome sequencing by sequence-independent single primer amplification (SISPA)

RNA was reverse transcribed into cDNA using primer K-8 N (5’ GAC CAT CTA GCG ACC TCC AC -NNNNNNNN 3′) at a concentration of 2.5 μM using the SSIV First strand cDNA synthesis system Kit (Invitrogen, USA) following the manufacturer’s instructions. Residual RNA was then removed by treating with RNase H for 20 min at 37°C. Next, double stranded (ds) cDNA was synthesized using DNA polymerase I, Large (Klenow) fragment (New England Biolabs, USA) in the presence of 800 nM of K-8 N primer by polymerization at 37°C for 60 min followed by inactivation at 75°C for 10 min. PCR amplification of ds cDNA was performed using a Pfu Ultra II Fusion HS DNA polymerase Kit (Agilent Technologies, USA) with 400 nM of primer K- (5’ GAC CAT CTA GCG ACC TCC AC 3′). The thermal cycling profile was initial denaturation of 1 min at 95°C followed by 45 cycles of 20 s denaturation at 95°C, 20 s of annealing at 55°C and extension of 3 min at 72°C, ending with a final extension of 3 min at 72°C. The amplified ds cDNA was purified using 1.8X AMPure XP beads (Beckman, USA).

The purified ds cDNA was then used for library preparation to run on an S5 Next generation sequencing system (Ion Torrent, Thermo Fisher). Approximately 50-100 ng of the ds cDNA were enzymatically fragmented to a length of 200 bp using Ion shear Plus reagents (Thermo Fisher Scientific, USA) with an optimized shearing time of 13 min followed by purification using 1.8X AMPure XP beads. The purified and fragmented DNA was ligated with adapters, barcodes to prepare barcoded libraries using an Ion Xpress™ Plus Fragment Library Kit and Ion Xpress barcode adapters (Thermo Fisher Scientific, USA). The libraries were purified using 1.2X AMPure XP beads and were then size selected on a Pippin Prep platform (Sage Science, Inc., USA) followed by a further purification step using 1.5X AMPure XP beads. The purified size selected libraries were subjected to 8 cycles of amplification using a Platinum™ PCR Super Mix High Fidelity kit (Thermo Fisher Scientific, USA) and purified using 1.2X AMPure XP beads. The purified amplified barcoded libraries were pooled at a concentration of 100 pM in equal volumes and loaded on to an Ion Chef™ instrument (Thermo Fisher Scientific, USA) for automated template preparation and chip loading using the Ion 540™ Kit-Chef (Thermo Fisher Scientific, USA). With Ion Chef™ the pooled libraries were clonally amplified on Ion Spheres™ (ISPs) by emulsion PCR followed by automated loading of template enriched ISPs on to an Ion 540™ chip (Thermo Fisher Scientific, USA). The sequencing was performed on an Ion S5 next generation sequencing system with 500 flows to generate 200 bp reads.

The raw sequences were cleaned to remove low-quality reads (Phred < 20), SISPA adapters, and short reads (<50 bp) using fastq-mcf v1.04.676 (ea-utils) and cutadapt v3.5. The quality of the reads was assessed with FastQC (v0.11.5). *De Novo* Assemblies were performed using Megahit (v1.2.9) and Spades (v3.15.5). Using the *De Novo* Assembly’s contigs, BLAST searches were performed, which identified complete genome (GenBank: MW057694), as the appropriate reference. After mapping the cleaned raw reads against the reference sequence using BWA (v0.7.17), SAMtools (v1.11) was used to generate Mpileup files and perform variant calling using BCFtools (v1.9), filtering only variants with a mapping quality > 20 and a minimum coverage depth of 100. The consensus sequences, from reads with a mapping quality > 20, were produced using vcfutils.pl. (VCFtools v0.1.16) and seqtk (v1.3.106) and compared to the *De Novo* assemblies. The mapping quality and coverage were assessed with Qualimap v2.3 (31). Additionally, the bam files were loaded into Integrative Genomics Viewer (IGV software version 2.3; The Broad Institute, Cambridge, MA) for read visualization.

### 3.5 Phylogenetic analysis

Phylogenetic trees using representative sequences from GenBank were estimated using the maximum-likelihood (ML) method available in MEGA 6 (21), employing the Tamura-Nei model of nucleotide substitution and 1,000 bootstrap replications.

### 3.6 Analysis of sub-consensus variants

To analyse the viral subpopulations in the cattle and dog samples, reads mapping to the targeted region with evidence of a mixed population (bp position: 11315–11,565 as suggested by the IGV visualization) were extracted from the sorted BAM file using Samtools, selecting only alignments with a mapping quality higher than 50 and supplying a BED file of the targeted region. The extracted reads were converted to FASTQ using Samtools’ bam2fq and trimmed with Cutadapt to keep only reads of lengths between 170 and 250 bp. Following the conversion of the reads from FASTQ to FASTA using Seqtk, the reads were aligned using MAFFT.

The MAFFT alignment was manually inspected to keep reads that were fully overlapping a 120 bp region of the targeted area and to filter out reads that were represented less than 3 times. Sequences from a similar region in publicly available rabies sequences were also retrieved from GenBank and merged with the dataset of the current study. Following a new alignment with MAFFT, only sequence variants that were represented 2 times or more were kept. Additionally, the sequences were translated into amino acids and reads that included truncated or incorrect amino acid sequences were excluded from further analysis. The final dataset consisted of 590 sequences.

### 3.7 Discriminant analysis of principal components (DAPC)

After manual inspections of the MAFFT-aligned sequences, the alignment file was imported into the ‘adegenet’ package in R for SNP analysis. Initially, the alignment file was converted into a ‘genlight’ object using the ‘fasta2genlight’ function, and the SNPs were extracted. The distribution and density of the SNPs among RABVs sequences were visualized using a heatmap. Additionally, discriminant analysis of principal components (DAPC) was conducted on the extracted SNPs to identify different clusters of RABVs, which were represented through PCA scatterplots.

## 4 Results

### 4.1 RDT and RT-PCR

The brain samples from both the puppy and the bull were positive by RDT and RT-PCR (See Supplementary Figure S2).

### 4.2 Full genome and phylogenetic analysis

Genomes of 11,886 bp, were resolved for D1, D2 and C2. The mean coverage for D1, D2 and C2 were 1823, 9,132 and 514, respectively. All the ORFs for the nucleoprotein (N), phosphoprotein (P), matrix protein (M), glycoprotein (G), and RNA-dependent RNA polymerase (L) were identified. In the 3′ and 5′ untranslated regions (UTRs) of the genomes, 23 and 14 nucleotides were unresolved, respectively.

**Figure 2:**
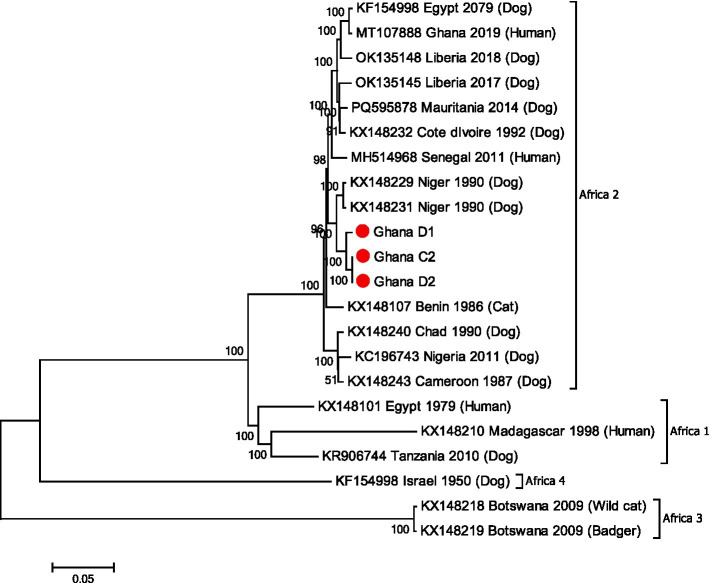
M-L phylogenetic tree generated using the genomes from the RABV positive samples and other RABV genomes available in GenBank (Supplementary Table S1). Comparative dog sample (D1), the puppy (D2) and bull sample (C2) are shown with red circles. Clades and bootstrap values from 1,000 replicates are indicated.

Phylogenetic analysis revealed that the genome sequences of D2 and C2 were identical to each other and that they clustered within the Africa-2 clade along with other RABVs from the region including Niger and Burkina Faso (Figure 2).

Further analysis of the sequences of C2 identified nucleotide differences in a sub-population of viral sequences (Supplementary Figure S3). Following the quality control of the region containing the SCVs there were 32 sequences available for alignment (see also Supplementary Figure S3). Of these 4 (12.5%) sequences contained the SCVs. These sub-consensus variants (SCV) were in the RNA-dependent-RNA polymerase (L) gene between region (bp 11,315–11,565) and were all synonymous mutations (Supplementary Figure S4).

### 4.3 PCA analysis

The PCA analysis of the genomic region where the SCVs were observed in 590 RABVs available in GenBank, revealed four clearly distinct clusters, with cluster 4 containing sequences of RABVs of African origin exclusively (Supplementary Figure S5). The consensus sequences of D2 and C2 were located in cluster 4. In contrast, the SCVs seen in the C2 sample were located in cluster 2 together with sequences from RABVs originating in Asia and North America.

## 5 Discussion

The devastating impact of rabies requires an expedited approach to handling outbreaks. Therefore, the speed with which an investigative team was assembled and dispatched to the site is commendable and this action averted a potentially devastating outbreak or spillover into humans. The case, however, highlights the inadequate knowledge of the public on bovine rabies. Thus, while rabies in the puppy was easily recognised, the virus in the bull was not. Perhaps, its rarity compared to canine rabies [only 1 recorded case in Ghana between 2010 and 2017 (14)] might explain the lack of public awareness of bovine rabies. The follow up public education was therefore an important aspect of the outbreak management.

Certain combinations of neurologic and generalized signs, albeit not pathognomonic, are highly suggestive of rabies. For instance, the drastic behavioral changes, *butter*-*like* hypersalivation, vocalization, confusion, gait asymmetry, dyspnoea and muscle tremors observed in the rabid bull have been associated with rabies (1, 5, 6, 11, 22–25). Depending on the site of viral introduction, the incubation of bovine rabies is between 8 and 30 days (23, 25). The short incubation observed in this outbreak could be attributed to the proximity of the puppy’s bite (on the muzzle) to the brain and, possibly, a high viral load.

The implicated RABV in this outbreak belonged to the Africa 2 lineage. The predominance of the Africa 2 lineage in West Africa had been attributed to free movement, trade and consumption of dogs within the sub-region (18). The availability of well-defined field samples in this study provided a unique opportunity to investigate the virus at the complete genomic level. The identification and analysis of variants within a viral population may identify nucleotides that have the potential to be selected if environmental conditions change (26) including adaptation to different hosts (27). Although, the consensus genome sequences of the RABV from the dog and bull samples in this current study were identical, SCVs were observed in the L gene of the RABV genome from the bull. Interpreting these SCVs is difficult given that they consisted of synonymous mutations that did not alter the amino acid sequence of the polymerase. However, PCA analysis indicated that the SCVs identified do not appear to be random or unique to the virus from this study since they have been identified in other RABVs albeit collected in different continents although there is no evidence of any epidemiological link between RABVs from Africa and those from Asia and the Americas. It is tempting to think that the SCVs might have become more dominant in the mixed viral population if the virus in the bull had more time to evolve. As it stood, the bull developed symptoms quickly due to the location of the bite and was destroyed within 4 days of being infected.

The responding team failure to screen other animals for rabies in the outbreak area was a major limitation of the on-site investigation. According to the investigators, this was due to the limited number of RDT kits available for screening. This lack of suitable diagnostic kits could have had serious public health implications due to the mixed species present in the livestock market (e.g., sheep, goat, and horse) and the limited experience of the general public with rabies presentation in these animals.

Livestock rabies has significant public health, economic and food security implications in many endemic countries; some of which have adopted livestock vaccination (10, 13, 28–30). Currently, Ghana neither vaccinates against nor prioritises livestock rabies possibly due to the cost of anti-rabies vaccines, and the relatively lower incidence of livestock rabies. Nevertheless, it will be beneficial for Ghana, like many other endemic countries, to consider an extensive public education on rabies, including bovine rabies.

## Data Availability

The datasets presented in this study can be found in online repositories. The names of the repository/repositories and accession number(s) can be found in the article/Supplementary material.
