# Supplementary material for: Case Report: Molecular characterization of rabies virus transmitted from a dog to a bull in a livestock market in Ghana
Source: Front Vet Sci. 2025 Jun 18;12:1524562. doi: 10.3389/fvets.2025.1524562 (PMC12213385; doi:10.3389/fvets.2025.1524562)
Supplement: Supplementary file 1 [file Data_Sheet_1.docx]

Supplementary Material


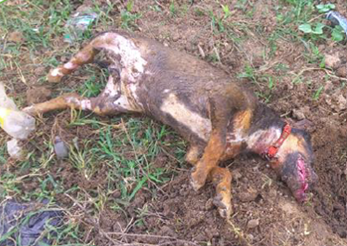
Supplementary Figure S1: Photograph of exhumed dog

MW D1 D2 C2 Neg Neg Pos


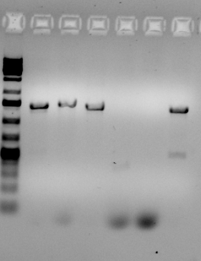


**Supplementary Figure S2: Gel image of RT-PCR amplification of RABV.**

MW: Molecular weight marker, Neg: negative control (nuclease free water), Pos: Positive RABV RNA. C2: bull sample, D1: dog sample R/24/19 D2: sample from puppy


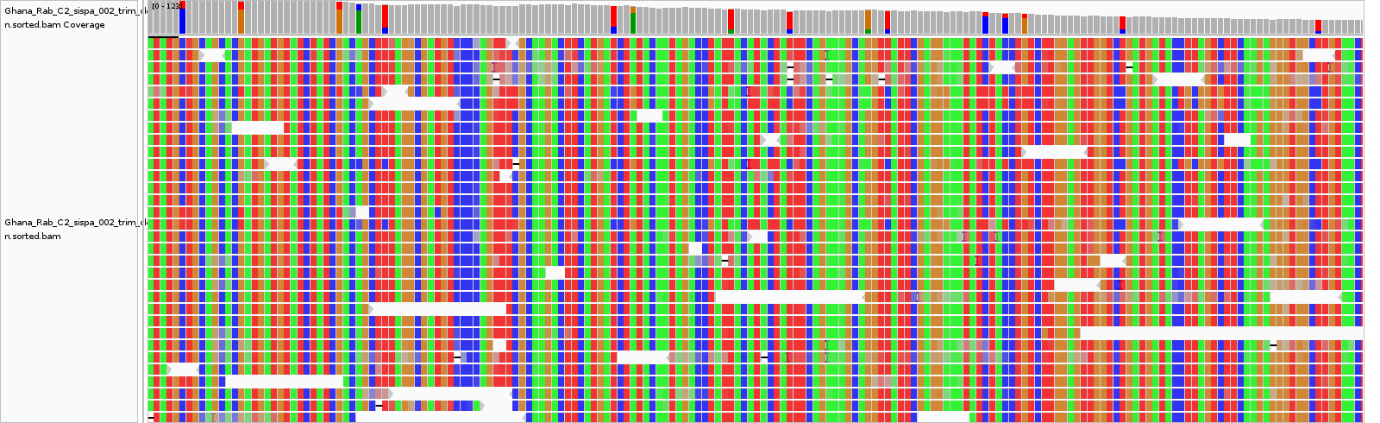


**Supplementary Figure S3** IGV visualization of the genome sequence (bp 11315 to11565) of the RABV from the bull samples (C2). SCVs are indicated by colour changes in the grey consensus sequence.

Ghana_Rab_C2vs atatg**T**tgcagca**T**gatgatgtatctatc**T**ac**C**gct**C**taggcgatgtccccagtttcgcaagactt

Ghana_Rab_C2 atatgctgcagcacgatgatgtatctatcgacagctttaggcgatgtccccagtttcgcaagactt

Ghana_Rab_D2 atatgctgcagcacgatgatgtatctatcgacagctttaggcgatgtccccagtttcgcaagactt

Ghana_Rab_D1 atatgctgcagcacgatgatgtatctatcgacagctttaggcgatgtccccagtttcgcaagactt

Ghana_Rab_C2vs catga**C**ct**G**tacaatagacctat**A**acatacta**C**ttcagaaagca**A**gt**C**attcgaggaaatat**T**ta**T**

Ghana_Rab_C2 catgatctatacaatagacctattacatactatttcagaaagcaggttattcgaggaaatatctac

Ghana_Rab_D2 catgatctatacaatagacctattacatactatttcagaaagcaggttattcgaggaaatatctac

Ghana_Rab_D1 catgatctatacaatagacctattacatactatttcagaaagcaggttattcgaggaaatatctac

Ghana_Rab_C2vs ct**T**tcttggagttggtc**C**gatgataccttagtcttcaagagggtggc**C**tgtaac

Ghana_Rab_C2 ctgtcttggagttggtctgatgataccttagtcttcaagagggtggcttgtaac

Ghana_Rab_D2 ctgtcttggagttggtctgatgataccttagtcttcaagagggtggcttgtaac

Ghana_Rab_D1 ctgtcttggagttggtctgatgataccttagtcttcaagagggtggcttgtaac

**Supplementary Figure S4** Alignments of a segment of L gene sequences generated in this study indicating the presence of synonymous SNPs. The SCVs from the bull sample are indicated in red


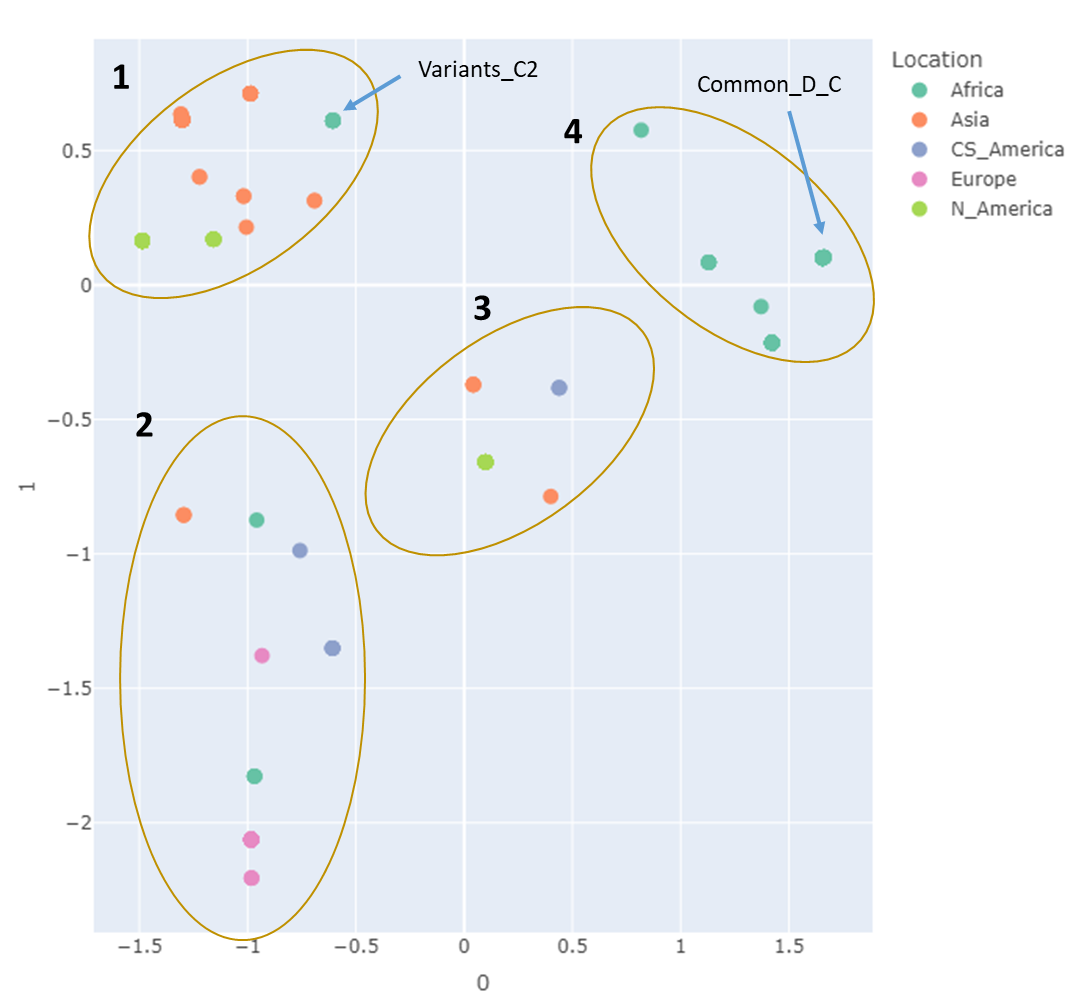


**Supplementary Figure S5.** PCA analysis based on the SNPs in the selected region (bp) of 590 RABV genomes. Clustered are circled and numbered 1 to 4.

**Supplementary video 1**

Supplementary video 1: circulated video of rabid bull

**Table S1** Genome sequences used in phylogenetic analysis

| **Accession number** | **Country** | **Year** | **Host** | **Clade** |
| --- | --- | --- | --- | --- |
| PP737682 | Ghana D1 | 2023 | Dog | Africa 2 |
| PP737683 | Ghana D2 | 2023 | Dog | Africa 2 |
| PP737684 | Ghana C2 | 2023 | Bull | Africa 2 |
| PQ595878 | Mauritania | 2014 | Dog | Africa 2 |
| KX148232 | Côte D’Ivoire | 1992 | Dog | Africa 2 |
| OK135145 | Liberia | 2017 | Dog | Africa 2 |
| KX148234 | Burkina Faso | 1986 | Dog | Africa 2 |
| OK135148 | Liberia | 2018 | Dog | Africa 2 |
| MT107888 | Ghana | 2019 | Human | Africa 2 |
| KX148229 | Niger | 1990 | Dog | Africa 2 |
| KX148231 | Niger | 1990 | Dog | Africa 2 |
| KX148107 | Benin | 1986 | Cat | Africa 2 |
| KX148240 | Chad | 1990 | Dog | Africa 2 |
| KC196743 | Nigeria | 2011 | Dog | Africa 2 |
| KX148243 | Cameroon | 1987 | Dog | Africa 2 |
| KX148210 | Madagascar | 1998 | Human | Africa 1 |
| KX148101 | Egypt | 1979 | Human | Africa 1 |
| KR906744 | Tanzania | 2010 | Dog | Africa 1 |
| KF154998 | Israel | 1950 | Dog | Africa 4 |
| KX148218 | Botswana | 2009 | Wild cat | Africa 3 |
| KX148219 | Botswana | 2009 | Badger | Africa 3 |
